# Supplementary material for: Chronic Inflammation Disrupts Circadian Rhythms in Splenic CD4+ and CD8+ T Cells in Mice
Source: Cells. 2024 Jan 13;13(2):151. doi: 10.3390/cells13020151 (PMC10814081; doi:10.3390/cells13020151)
Supplement: Supplementary file 1 [file cells-13-00151-s001.zip › cells-2788526-supplementary.pdf]

Title:

Chronic Inflammation Disrupts Circadian Rhythms in Splenic CD4+ and CD8+ T Cells in Mice

Authors:

Misa Hirose[1,2,3], Alexei Leliavski[4], Leonardo Vinícius Monteiro de Assis[1,2], Olga Matveeva[1], Ludmila Skrum[1,2], Werner Solbach[5], and Henrik Oster[1,2,#], Isabel Heyde[1,2,#]

Affiliations:

[1]: Institute of Neurobiology, University of Lübeck, Lübeck, Germany

[2]: Center of Brain, Behavior and Metabolism, University of Lübeck, Lübeck, Germany

[3]: Institute of Experimental Dermatology, University of Lübeck, Lübeck, Germany

[4]: T-knife GmbH, Berlin, Germany

[5]: University of Lübeck, Lübeck, Germany

[#]: correspondence to HO: henrik.oster@uni-luebeck.de; tel. +49-451 3101 4300 / IH: is.heyde@uni-luebeck.de; tel. +49-451 3101 4312, Ratzeburger Allee 160, 23562 Lübeck, Germany

Supplemental tables

Table S1: Primers used for qPCR.

Gene	Forward (5'→3')	Reverse (5'→3')
Bmal1	CCTAATTCTCAGGGCAGCAGAT	TCCAGTCTTGGCATCAATGAGT
Dbp	AATGACCTTTGAACCTGATCCCGCT	GCTCCAGTACTTCTCATCCTTCTGT
Eef1a	TGCCCCAGGACACAGAGACTTCA	AATTCACCAACACCAGCAGCAA
Foxp3	CCAGTACTCAGGGCAGTGT	GTGGAAGAACTCTGGGAAGG
Gata3	TATGTGCCCGAGTACAGCTC	CTCCCTGCCTTCTGTGCT
Gzmb	GCCCACAACATCAAAGAACAG	AACCAGCCACATAGCACACAT
Ifng	AGCTCTTCCTCATGGCTGTT	TTTGCCAGTTCCTCCAGATA
Il10	CCTGGTAGAAGTGATGCCCC	TCCTTGATTTCTGGGCCATG
Nr1d1	AGCTCAACTCCCTGGCACTTAC	CTTCTCGGAATGCATGTTGTTC
Per1	AGTTCCTGACCAAGCCTCGTTAG	CCTGCCCTCTGCTTGTCATC
Prf1	AGCCCCTGCACACATTACTG	CCGGGGATTGTTATTGTTCC
Rorc	CAGAGGAAGTCAATGTGGGA	ATGATCTGGTCATTCTGGCA
Tbx21	AGCAAGGACGGCGAATGTT	GGGTGGACATATAAGCGGTTC

Table S2: p-values for cosinor and amplitude analysis.

		Cosinor			Amplitude (vs. CON)	
T cell population	Gene	CON	CFA/PTx	EAE	CFA/PTx	EAE
CD4+	Number	0.1658	0.1522	0.0031	0.8429	0.7805
	Bmal1	0.0039	0.0912	0.0111	0.6068	0.1946
	Per1	0.0156	0.0001	0.3033	0.2240	0.1242
	Nr1d1	0.0299	0.0079	0.0453	0.4724	0.8099
	Dbp	0.0004	0.2623	0.1194	0.0003	0.0156
	Gata3	0.0002	0.3573	0.8883	0.9481	0.0330
	Foxp3	0.0016	0.4863	0.6791	0.3079	0.0332
	Il10	0.2420	0.2031	0.6036	0.6957	0.6739
	Ifng	0.1668	0.3465	0.8050	0.7900	0.5723
	Rorc	0.4247	0.2241	0.1770	0.4230	0.3056
	Tbx21	0.0620	0.0828	0.6326	0.3807	0.5193
CD8+	Number	0.1417	0.0867	0.5715	0.9098	0.6309
	Bmal1	0.3404	0.1574	0.2365	0.3637	0.6500
	Per1	0.0348	0.0000	0.2680	0.5093	0.2067
	Nr1d1	0.2143	0.0038	0.0609	0.3532	0.8812
	Dbp	0.0003	0.0282	0.0536	0.0292	0.0104
	Prf1	0.0867	0.0395	0.1256	0.1001	0.5671
	Gzmb	0.0876	0.1850	0.3427	0.9776	0.7862
